# Magnetic Field Feature Extraction and Selection for Indoor Location Estimation

**DOI:** 10.3390/s140611001

**Published:** 2014-06-20

**Authors:** Carlos E. Galván-Tejada, Juan Pablo García-Vázquez, Ramon F. Brena

**Affiliations:** Instituto Tecnologico de Monterrey, CETEC South Tower, 5th floor, Avenue. E. Garza Sada 2501, 64849, Monterrey NL, Mexico; E-Mails: jpablo.garcia@itesm.mx (J.P.G.-V.); ramon.brena@itesm.mx (R.F.B.)

**Keywords:** mobile sensors, magnetometer, location, indoor positioning, location estimation, feature extraction, feature selection, magnetic field measurement

## Abstract

User indoor positioning has been under constant improvement especially with the availability of new sensors integrated into the modern mobile devices, which allows us to exploit not only infrastructures made for everyday use, such as WiFi, but also natural infrastructure, as is the case of natural magnetic field. In this paper we present an extension and improvement of our current indoor localization model based on the feature extraction of 46 magnetic field signal features. The extension adds a feature selection phase to our methodology, which is performed through Genetic Algorithm (GA) with the aim of optimizing the fitness of our current model. In addition, we present an evaluation of the final model in two different scenarios: home and office building. The results indicate that performing a feature selection process allows us to reduce the number of signal features of the model from 46 to 5 regardless the scenario and room location distribution. Further, we verified that reducing the number of features increases the probability of our estimator correctly detecting the user's location (sensitivity) and its capacity to detect false positives (specificity) in both scenarios.

## 1. Introduction

Identifying the location of a user in an indoor environment has been the focus of many research groups (e.g., IndoorAtlas Ltd. [1], CommLAB [2], WRC [3], DraMCo [4], NAVVIS [5], CEIT [6]. This is due to the emergence of a large number of systems that aim to offer services to users based on their location.

In order to estimate the user indoor location, several technological approaches have been proposed to develop indoor location systems (ILS), for instance, using RFID, WiFi, Bluetooth, Ultra Wide-band (UWB), computer vision, ultrasonic sensors, among others [7–11]. These approaches can estimate the user location with an acceptable accuracy. However, they present disadvantages. Most of them require a dedicated infrastructure (e.g., access points, array of sensors or cameras), thus the system scalability can be expensive, as it requires adding devices. Besides, their coverage is limited since it depends on the infrastructure. Finally, in some cases, there is a high processing demand (e.g., computer vision). Nevertheless, sometimes it is possible to deploy such technologies reusing existing infrastructure [12–14]. For instance, Vera *et al.* [13] propose the use of WiFi signals to develop an ILS without having to deploy extra infrastructure. They calculate the signal power of each available WiFi through a propagation model; however, the need to have WiFi covered areas reduces the scalability of the ILS to places that are covered by the signal. New technological approaches have been proposed to tackle these issues; for instance, approaches using the signals that are already available in the indoor environment, such as magnetic field [14–18].

The main idea of these works is to use the irregularities of the earth's natural magnetic field induced by the building's structures and other elements common in indoor environments, and to detect these irregularities as clues for finding the user's location, with the help of a magnetometer such as those available in commercial smartphones. These approaches require mapping a given indoor environment beforehand, measuring at each point the magnitude and direction of the magnetic field, and then, using this magnetic map for location purposes, finding the most similar place in the magnetic map to the one detected at a given point.

In our approach, the goal is to identify the “room” in which the user resides at a certain moment. Unlike some other methods that strive to determine the exact coordinates, we contend that in most practical situations, instead of a vector of coordinates, to know in which room the user is located is exactly the type of information needed.

The contributions of this paper are two-folds:

(1) We developed an original method that relieves the need to construct a detailed magnetic map—a grid of magnetic measures for each point in the building—as other approaches require. Instead, we just store a kind of “signature” taken from a random walk inside a given room, which takes as an essential component the frequency spectrum of the magnetic signal, obtained from the Fourier transform of that signal. This method proves to be independent of the exact path used when picking the magnetic signal, thus giving it very desirable robustness.
(2) We present an extension and improvement of our previous indoor localization model based on the feature extraction of 46 magnetic field signal features to estimate the location of a user in an indoor environment [19]. We describe our methodology in detail and we extend our experiments presented in Galván-Tejada *et al.* [19,20]. One of our aims is to reduce the amount of data required, because it has been developed with the purpose of being used in mobile devices, which have limited computational resources, compared with regular computers. The extension consists in adding a feature selection phase for reducing redundant features or inverse correlated data that can be embedded. In order to achieve it, a genetic algorithm (GA) is used as a feature selection algorithm to finally get a model to estimate the location. In addition, we provide evidence that our ILS method can estimate the user indoor location by evaluating it in two different real scenarios: house and an office building. To test the ILS model, two kinds of mobile device were used: smartphone and tablet.

This paper is organized as follows. After this introduction, our location methodology is described in Section 2. In Section 3 we present the experiments and results. A discussion of our results is presented in Section 4. Finally, our conclusions and future work are presented in Section 5.

## 2. Indoor Location Estimation Methodology

**Figure 1.**
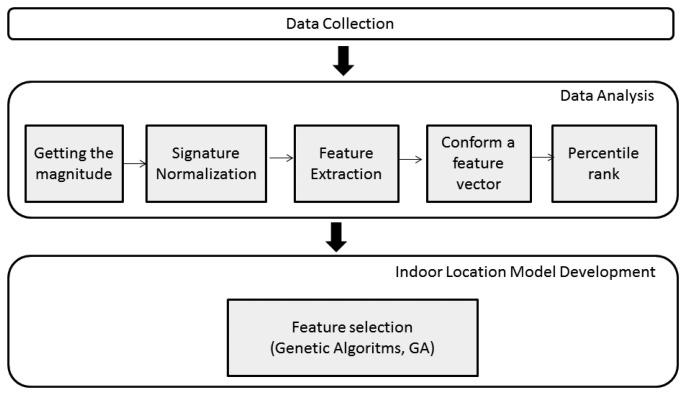
Indoor location methodology.

In this section, we present an extension and improvement of each step of our methodology for estimating user location using earth magnetic field, which was previously presented in Galávn-Tejada et al. [19,20]. This methodology consists of three phases, as is shown in Figure 1.

### 2.1. Phase 1: Data Collection

This phase collects the magnetic field information of an indoor environment. To collect data, the user must use our mobile application, which retrieves information from the magnetometer of a mobile phone, and walk around with the mobile phone in the given indoor environment for 10 s with an approximate speed of 1 m/s in order to cover the entire room. We call this basic set of data the signatures that represent a room. The mobile phone can be positioned on the user's waist or held on the user's hand.

In order to estimate the number of signatures needed to create a model for estimating the user indoor location, Equation (1) proposed by *Eberhardt* [21] was used. With this equation, we determined the minimal number of experiments (signatures) required, with the aim of having a statistical validation. In Equation (1), *x* is the minimum number of experiments, and *N* is the number of variables.

(1) $$x={\text{log}}_{2}(N)+1$$

### 2.2. Phase 2: Data Analysis

This phase consists of five activities described below.

(1) *Activity 1: Getting the Magnitude*. The collected magnetic field measures are modeled as a vector of three components *B_x_*, *B_y_*, and *B_z_* [17]. We can compute the total magnitude of the magnetic field using Equation (2), where *M_x_*, *M_y_*, and *M_z_* are the three physical axes along *x*,*y*, and *z* respectively. Using only the magnitude of the signal makes irrelevant the exact position that the user carries the magnetometer (that is, the tablet or smartphone), thus overcoming a requirement present in other proposals.
  (2) $$\left|M\right|=\sqrt{M_{x}^{2}+M_{y}^{2}+M_{z}^{2}}$$
(2) *Activity 2: Signature Normalization*. After obtaining the magnetic field magnitude, we eliminate spatial scaling and shifting by normalizing each signature using Equation (3), where *z_i,d_* is the normalized reading, *r_i,d_* refers to the *i^th^* observation of the signature in dimension *d, μd* is the mean value of the signature for dimension *d*, and *σ_d_* is the standard deviation of the signature for dimension *d*.
  (3) $$\forall i\in m:z_{i,d}=\frac{r_{i,d}-\mu_{d}}{\sigma_{d}}$$
  Equation (3) is applied for all dimensions in *R^d^*.
(3) *Activity 3: Feature Extraction*. This process extracts the minimal number of signal features that enables us to characterize its behavior. To select the appropriate set of features, we carry out a review of literature in digital signal processing (DSP) and from first and second order statistic parameters [22–25]; as a result we identify 46 features shown in Table 1, where 16 are from temporal domain and 30 are from spectral domain. *Temporal features* are computed from the waveform of magnetic field signal, while *Spectral features* are acquired performing a P-point Fast Fourier Transform to each signature of the magnetic field signal [26]; to achieve it, we apply Equation (4), where *ES_i_* is the *i^th^* energy signature of the normalized magnetic field signal, and *NS_i_* is the *i^th^* normalized signature.
  The first 10 components were extracted from the spectral evolution, because from observation we presume that the phenomena tend to be chaotic, so we assume that most of the energy is concentrated in these 10 first components.
  (4) $$\forall i\in n:ES_{i}=FFT(NS_{i})$$
(4) *Activity 4: Merging Features*. Once all the features are computed, all of them are merged into a dataset of features that summarizes the behavior of the signal, reducing the amount of data from 1000 data points to 46 per signature.
(5) *Activity 5: Percentile Rank*. To keep all the features in a range of 0 to 1 and ensure that they have the same impact in the model development, a percentile rank is applied using Equation (5), where *x* is the *x^th^* feature.
  (5) $$PR=\frac{\text{trunc}(\text{rank}(x))}{\text{length}(x)}$$

#### 2.2.1. Phase 3: Indoor Location Model Development

To get a model that allows the estimation of user location with fewer magnetic field signal features, a feature selection process through a Genetic Algorithm (GA) is applied.

A feature selection process can be viewed as an optimization problem; in this particular case the fitness of the model must be optimized with the minimum number of features. The Genetic Algorithm (GA) is a well-known method for solving this kind of problems. Other feature extraction techniques like Principal Component Analysis (PCA) and Singular Value Decomposition (SVD) rely too much on the variance of features extracted from data and tend to discard some features that are actually very helpful for the development of a location-finding system based on our methodology.

## 3. Experiments and Results

In this section, we present the experiments and results of each of the phases of the methodology presented in Section 2.

### 3.1. Data Collection

**Figure 2.**
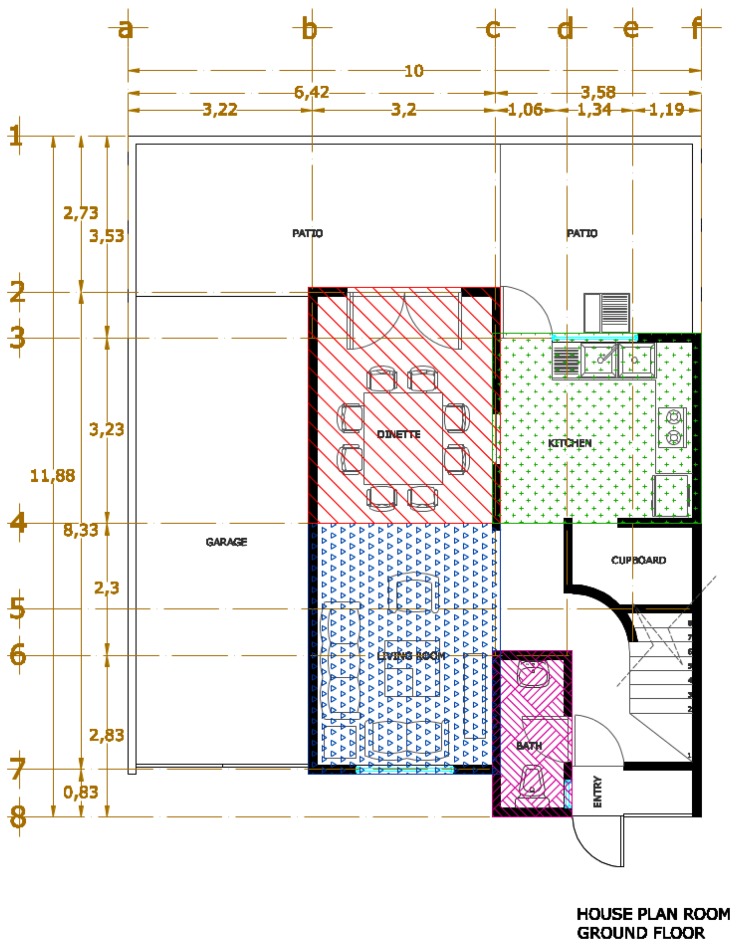
First floor house plans with furniture.

**Figure 3.**
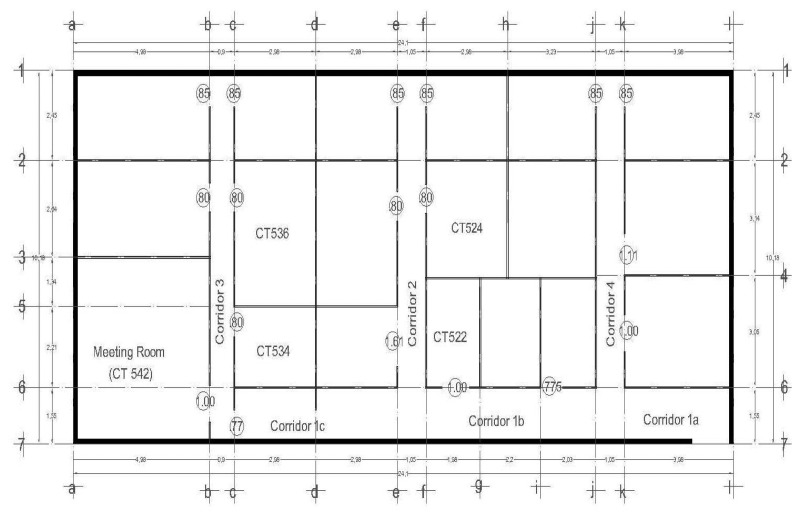
Office building layout.

We collect data in two scenarios: (i) a residential home that consists of 4 rooms: living room, dining room, kitchen and bathroom, as shown in Figure 2; (ii) an office building that consists of 20 rooms among which only eleven are considered, as shown in Figure 3.

Data were collected with a mobile application developed with Java using the Google API Level 7. This application is able to get information from magnetic field using the magnetometer of a mobile device. In our experiments, we used different mobile devices in different scenarios. In the residential home environment, we used a Samsung S3 i9300 smartphone with the official Samsung Android compilation 4.1 and a magnetic sensor model AKM8975, and also a tablet Acer 500 with the same magnetic sensor model. In the office building environment, we used a Samsung S4 i9505 with Android 4.3 compilation and a magnetic sensor model YAS532.

To determine the quantity of data that must be collected to generate a location estimation model for both scenarios, we used Equation (1) presented in Section 2.1.

For the residential home scenario, 9 signatures per room were taken, the number of signatures was calculated with the Equation (1); in this experimentation *N* is equal to 46, which is the number of features, multiplied by 4, the number of rooms; from that equation we obtain 8.52 signatures, which was then rounded to 9. To validate the model with a *k*-folds strategy, the number of signatures was increased to 10. For the office scenario, *N* is equal to 46, which is the number of features, multiplied by 11, the number of rooms; from that equation we obtain 9.9 signatures, which was then rounded to 10. In this particular case, the dataset was increased to 12 signatures in order to allow the *k*-folds strategy to be applied with a larger dataset.

All data collected in our experiments is available on the AAAMI research group website [27].

### 3.2. Data Analysis

Data analysis was done by programming a script in R [28], a free multi-platform software (GNU project) environment for statistical computing. The result of this phase was a data frame composed by 40 rows (10 per room) with 46 columns (features) in the residential home scenario, and 132 rows with 45 columns in the office building scenario. All features range between 0 and 1 after the percentile rank.

### 3.3. Indoor Location Model Development

**Figure 4.**
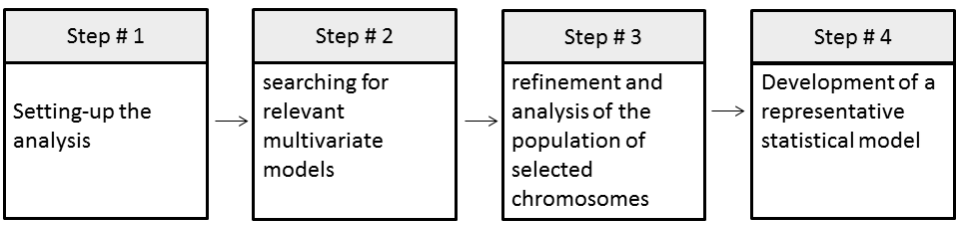
Galgo procedure.

This is the third phase of our location methodology, in which the Genetic Algorithm (GA) strategy is required. The Galgo R package [29] was used to solve the optimization problem. In this package, four steps are performed, as shown in Figure 4 and described in the following.

(1) *First Step: Setting-up the analysis*. The analysis using Galgo starts by pre-processing the data where the user specifies classes, variables and GA parameters in order to comply with the requirements of Galgo. All these processes can be done using other R tools. Finally the parameters that define the GA search environment are defined. For this experiment we define as classes the rooms names—that is, in the house environment shown in Figure 2: kitchen, dining room, bathroom and living room; in the office environment shown in Figure 3: CT542, CT536, CT534, CT522, CT524, Corridor 1a, 1b, 1c, Corridor 2, Corridor 3, and Corridor 4. The variables were the features extracted (Table 1) from the signatures, and finally 300 generations and a size of 5 for chromosomes were defined. These number or generations were chosen to cover a big number of combinations of features, and the chromosome size was selected to minimize the “curse of dimensionality”.
(2) *Second Step: Searching relevant multivariate models*. An evolutionary cycle begins from a random population of chromosomes of size predefined in the first step, in the parameters of the GA. In this case, we start 300 evolutionary cycles with the same configuration to expand even more the number of combinations.
(3) *Third Step: Refinement and analysis of population for selected chromosomes*. The GA procedure selects the chromosomes that have the desired classification accuracy. In this step, after the selection, an analysis of the genes can be done to reduce the possibility of having genes that do not contribute to the fitness of the model.
(4) *Fourth Step: Selection of a representative statistical model*. During all the processes of Galgo, several models are generated and in the final step, the best model is chosen.

**Figure 5.**
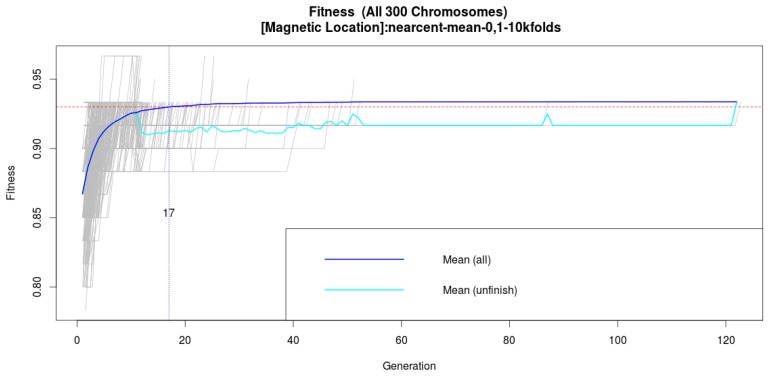
Fitness of the models through the evolutionary process.

The two last steps are shown in Figure 5 for residential home scenario. Each gray line represents the evolution of one chromosome, while the dark blue line represents the average fitness of all chromosomes. In this experiment, in order to maintain a short development time for the model, we propose a minimum fitness of 93 to achieve (red line), which indeed is a high accuracy of the final model of an ILS, instead of 100 that severely increases the time of the GA process.

**Figure 6.**
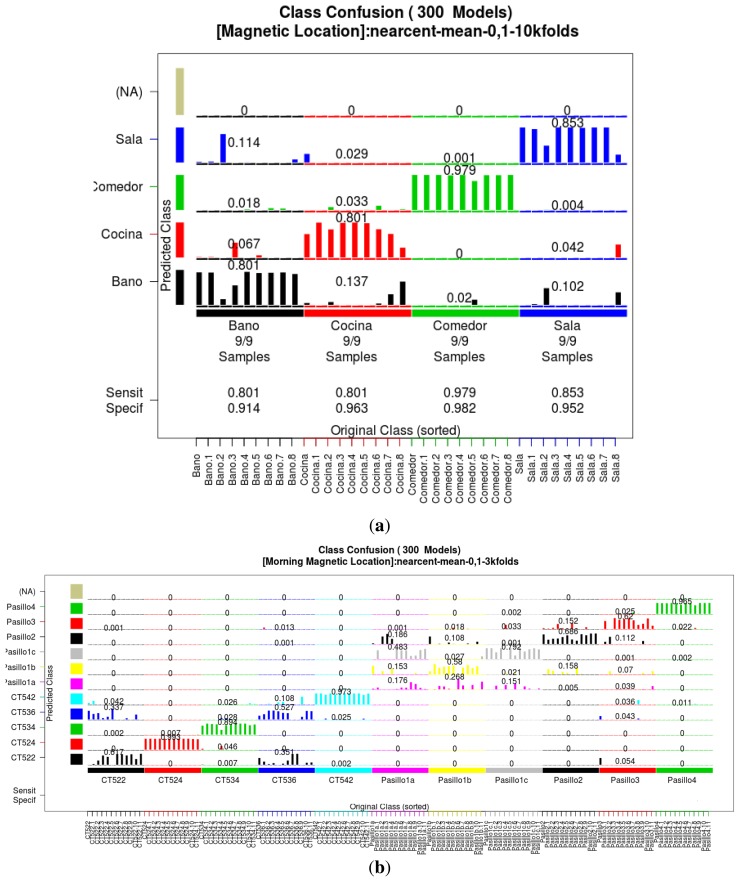
Confusion plot of the models; (**a**) residential home scenario; (**b**) office building scenario.

In order to validate the models, a leave-one-out strategy was used to ensure independence from the dataset that was used to train the model and prevent overfitting. This strategy allows us to know the behavior of the models in an unknown dataset; for instance when the model is used in real time in an ILS. Figure 6a presents the confusion matrix of the acquired classification model for the residential scenario after the GA process, and Figure 6b shows the confusion matrix of the model for the office scenario, which demonstrates the behavior of the model in a more complex indoor environment. In this experiment we calculated *sensitivity* as the probability of our estimator to detect correctly the location of the user, and *specificity* as the capacity of our estimator to detect false positives. We chose these measures instead of accuracy because we wanted more detailed parameters to measure the correct estimation of the location and avoid a high accuracy term filled with biased false positives. For both scenarios, on average, we obtained a sensitivity of 0.784 and specificity of 0.961, which are respectively the probability of our estimator to detect correctly the location of the user (sensitivity), and the capacity of our estimator to detect false positives (specificity).

**Figure 7.**
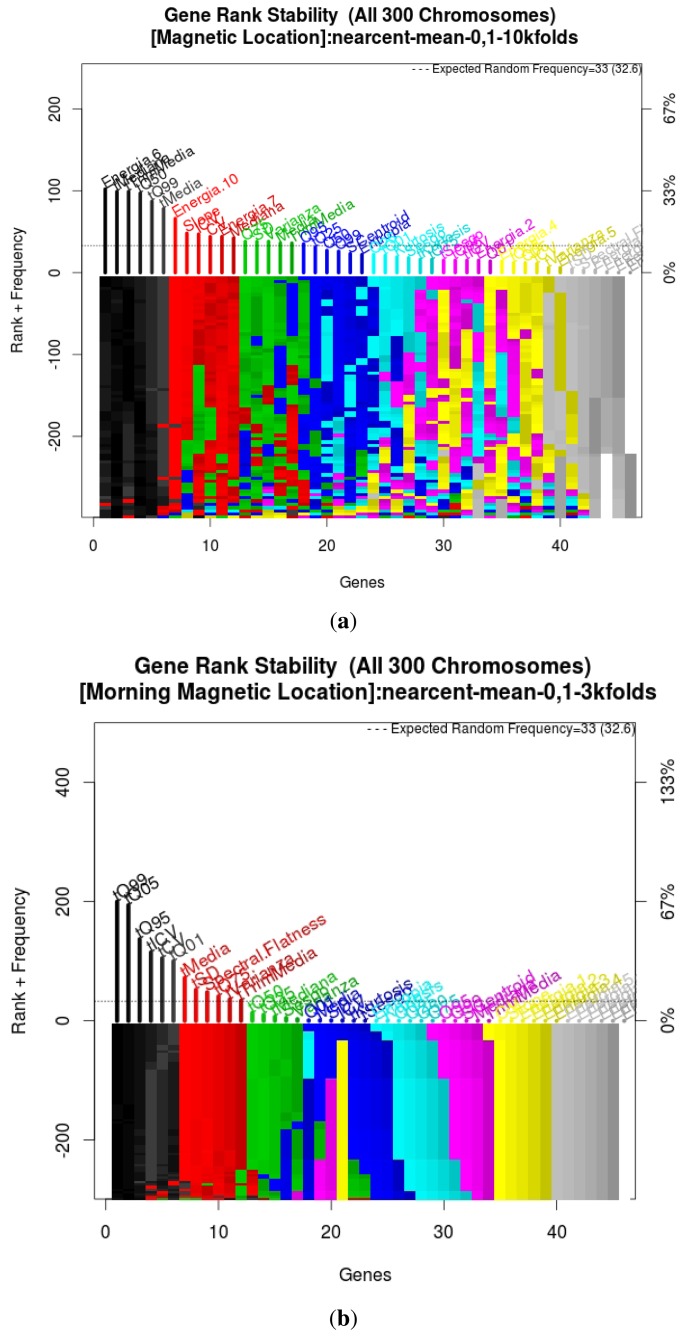
Feature ranking during GA; (**a**) residential home scenario; (**b**) office building scenario.

During the GA process, features were ranked according to their importance with respect to the location accuracy. Figure 7a,b shows the ranking of features for both scenarios, and we can see how most of the information is contained in just five features, the ones that compose the final model. Additionally, we can observe that temporal quantiles are present as features in the final model in both scenarios, which means that descriptive information is embedded in these features independently of the scenario and device.

## 4. Discussion

In this research, we focus on extending our previous indoor location estimation methodology with a phase of feature selection (FS) and evaluating it in two different scenarios: residential home and building office. The results presented in Section 3 allow us to identify the following aspects:

- *Applying a feature selection process enables us to increase the specificity and sensitivity of the ILS*. The results presented in Section 3 provide evidence that in comparison with our previous model, performing a feature selection process helps us to reduce by 90% the amount of information required to develop an indoor location system, as well as to increase specificity and sensitivity. For instance, we have identified in the residential home scenario an increase of 7.5% in the *sensitivity* and a 2.1% increase in the *specificity* of the system. Table 2 shows the comparison between the 46 features model and the 5 features model for both scenarios.
- *Temporal shape features are the most appropriate ones to characterize the behavior of the magnetic field signal*. In our results we identify that the surviving features after applying GA were taken from temporal evolution (see Section 3.3). This fact allows us to know that the FFT process and the feature extraction from the spectra can be avoided altogether, reducing the computational cost during the feature extraction phase of our methodology without decreasing the accuracy of the final model.
- *ILS Methodology can be used to create an indoor location model for estimating relative position in small indoor areas such as a residential home or big-size indoor area such as a building office*. As we presented in Section 3, we tested our ILS model in two different scenarios with different number of rooms. This demonstrates the behavior of the methodology in big scenarios (e.g., the office building) and the small scenarios (e.g., residential home). Our results indicate that the methodology is applicable regardless of the size of the location. However, if the space is expanded, in order to identify the new rooms, the model must be calibrated again; nevertheless, if the space is reduced while keeping the same room distribution, the model is still applicable.
- *Using statistical features to create an ILS model helps us to avoid problems related to magnetic field weakness*. We are aware of the weakness of the magnetic signal, as well as its dependence on the construction materials of the indoor location and the apparent lack of information embedded in it [30,31]. However, we show in this paper that using statistical features to model the behavior of the signal, instead of using just the raw signal, allows us to develop an ILS based on magnetic field features regardless of the size of the indoor location as well as the specific sensor for taking the measures. For instance, in our experiment we used three different devices, one Acer table 500 and two Samsung smartphones, and all gave the same results.

## 5. Conclusions and Future Work

The main contribution of this paper is the extension of our original method to develop an indoor location model. This extension is based on magnetic field feature extraction from magnetic field signatures, which is improved through a feature selection process using a GA approach. This methodology works regardless of the device used and the indoor environment size, distribution and construction material.

The inclusion of the feature selection process allows us to reduce the amount of required information to just 5 out of the original 46 features. The resulting model can be run on a standard smartphone that bears a magnetometer sensor.

As future work we plan to improve the current classification model. We may use stepwise regressions to remove the remaining highly correlated features (redundant information) that can survive along the GA process using the rank of features generated. To avoid the stochasticity and time consuming of the GA feature selection, we may also use Integrated Discrimination Improvement (IDI) and Net Reclassification Improvement (NRI) techniques. Additionally, once the model is refined, we have the hypothesis that a Markov Process may be adapted to improve the performance of the ILS, given the nature of the indoor location problem, the places and the transitions. In particular, the transitions from one room to another room are bounded by connectivity restrictions and can be taken into account.

## Figures and Tables

**Table 1.** Features extracted.

Features	Temporal Domain	Frequency Domain
Kurtosis	*	*
Mean	*	*
Median	*	*
Standard Deviation	*	*
Variance	*	*
Coefficient of Variation (CV)	*	*
Inverse CV	*	*
1,2,3 Quartile	*	*
1,5,95,99 Percentile	*	*
Trimmed Mean	*	*
Shannon Entropy		*
Slope		*
Spectral Flatness		*
Spectral Centroid		*
Skewness		*
1–10 Spectrum Components		*

**Table 2.** Comparison of the 46 and 5 features residential home model.

Approach	Sensitivity	Specificity
46 Features Model	0.783	0.934
5 Features After FS	0.858	0.952
